# Increased Risk for Infections and Allergic Disease in Hereditary Hemorrhagic Telangiectasia

**DOI:** 10.3390/jcm13133752

**Published:** 2024-06-27

**Authors:** Freya Droege, Jochem König, Karl S. Lang, Jadwiga Jablonska, Ekaterina Pylaeva, Carolin Huckenbeck, Anna Wrobeln, Inga Duerig, Kruthika Thangavelu, Stephan Lang, Urban Geisthoff

**Affiliations:** 1 Department of Otorhinolaryngology, Head and Neck Surgery and VASCERN HHT Reference Centre, Essen University Hospital, University Duisburg-Essen, Hufelandstrasse 55, 45147 Essen, Germany; jadwiga.jablonska@uk-essen.de (J.J.); ekaterina.pylaeva@uk-essen.de (E.P.); carolinlueb@googlemail.com (C.H.); inga.duerig@web.de (I.D.); stephan.lang@uk-essen.de (S.L.); 2 Institute of Medical Biostatistics, Epidemiology and Informatics, University Medical Center of the Johannes Gutenberg-University Mainz, 55101 Mainz, Germany; koenigjo@uni-mainz.de; 3 Institute of Immunology, Essen University Hospital, University Duisburg-Essen, Hufelandstrasse 55, 45147 Essen, Germany; karlsebastian.lang@uk-essen.de; 4 Institute of Physiology, Essen University Hospital, University Duisburg-Essen, Hufelandstrasse 55, 45147 Essen, Germany; anna.wrobeln@uk-essen.de; 5 Department of Otorhinolaryngology, Head and Neck Surgery and VASCERN HHT Reference Centre, University Hospital of Marburg, Philipps-University of Marburg, Baldingerstrasse, 35042 Marburg, Germany; kruthika.thangavelu@gmail.com (K.T.); geisthof@med.uni-marburg.de (U.G.)

**Keywords:** allergies, hereditary hemorrhagic telangiectasia, infections, pulmonary arteriovenous malformation

## Abstract

**Background/Objectives**: Hereditary hemorrhagic telangiectasia (HHT) is a rare disorder characterized by dilated blood vessels. Different immunological changes have been described in these patients. In this study, the predisposition of patients with HHT to infections and allergic diseases was assessed. **Methods**: Patients with HHT completed an online survey in English or German. Their data were compared to non-affected partners or friends. **Results**: A total of 430 out of 588 respondents with HHT answered our questions about infections and allergies. Patients with HHT suffered significantly more often from various types of allergies than their partners, especially type I allergies (n = 226/276, 82%), and had a higher risk for sinusitis, urinary tract infections, pulmonary infections, and abscesses. A total of 38% of the patients took antibiotics prior to dental or surgical procedures (n = 57/152), and, in 10% of these patients, pulmonary arteriovenous malformations (PAVMs) were not detected. On the other hand, 51% of patients with PAVM did not report a prophylactic antibiotic intake (n = 40/79). The patients who needed iron supplementations suffered more often from sepsis (OR: 9.00, 95%CI: 0.92–88.16). **Conclusions**: Compared to their non-affected controls, patients with HHT showed an increased risk for infections in different organs and allergic diseases. There is a need for campaigns raising greater awareness recommending prophylactic antibiotic intake in patients with PAVM.

## 1. Introduction

Hereditary hemorrhagic telangiectasia (HHT) is an inherited rare disease with arteriovenous malformations occurring systemically. Patients with this disease suffer from recurrent bleeding, especially from the nose and gastrointestinal tract, and visceral vascular malformations particularly affecting the brain, liver, and lungs [1,2]. Using the clinical Curaçao criteria, the diagnosis of HHT can be confirmed [3]. Mutations in several genes of the transforming growth factor beta (TGF-β) pathway have been found in patients with HHT. Most frequently, endoglin (ENG, HHT Type 1) or the activin receptor-like kinase-1 (ACVRL1 or ALK1, HHT Type 2) are affected in HHT [4,5,6].

Previous studies suggested alterations in the immune cells, leading to an increased susceptibility to bacterial infections in HHT patients. Severe changes in the innate and adaptive immune system of patients with HHT have been detected, such as a reduction in neutrophils and monocyte function and the ablation of T helper cells [7,8,9]. Monocyte differentiation and phagocytotic processes are influenced by endoglin. Therefore, mice lacking this gene have been shown to be prone to spontaneous infections [10]. Furthermore, in patients with pulmonary arteriovenous malformations (PAVMs), their lung filtering capability is bypassed, leading to a potential formation of paradoxical embolism. Embolisms and subsequent bacterial seeding or primary septic embolism may then result in developing potentially fatal brain abscesses. Thus, screening for PAVM is recommended in every person with (suspected) HHT [11,12].

In HHT, recurrent bleeding may lead to severe anemia, which may require treatment with blood transfusions. Transfusion reactions are rare and may range in severity from mild to life-threatening. The risk of developing a transfusion reaction increases with the number of transfusions given [13]. Moreover, immunoglobulins are important mediators in type I and type II allergies. Altered immunoglobulin levels have been reported in HHT [7,14].

The aim of this study was to determine whether the frequency of infections and allergies in patients with HHT was altered and assess the antibacterial treatment they received. Knowledge of these data might improve patient management. 

## 2. Materials and Methods

We designed an online questionnaire in German covering several steps, including a discussion at an annual meeting of the national German HHT self-help group. Afterwards, two native bilingual speakers (one patient with HHT and one physician) helped to translate the survey into English, and several international self-help groups distributed it (see Acknowledgements). The survey included questions about the clinical Curaçao criteria, the general medical history of HHT (questions 4–48 in the Supplementary Materials), different allergies (hay fever/asthma, drug/food allergy, contact allergy), and diverse infections and their treatment (questions 119–134 in the Supplementary Materials). The diagnosis of HHT could be established using the criteria published elsewhere (see Supplemental Materials S1 and References [15,16]).

The Epistaxis Severity Score (ESS) [17] was calculated in relation to the previous four weeks and used to categorize the patients’ epistaxis (see Supplementary Materials S2). Results from the open survey were collected from February 2016 to August 2018. An average hemoglobin level below 12.0 g/dL in women and below 13.0 g/dL in men was defined as anemic [18]. Descriptive statistics (number/percentage of patients (n, %) and mean ± standard deviation (m ± SD)) were used for the general history of HHT and geographical data of the patients. Different patient groups were compared using two-sample t-tests, an odds ratio (OR) with 95% confidence intervals (CIs), and chi-square tests (χ^2^). Each patient with HHT was asked to compare different aspects such as the frequency of suffering from different types of infections or the quantity of antibiotic intake with their non-affected partner or friend. For analyzing these paired data, we used the McNemar test. The ratios of odds with 95% confidence intervals were calculated from patients with HHT who suffered more or less often from different infections compared to their non-affected partner or friend. Statistical analyses were performed with IBM SPSS Statistics (version 26, Armonk, New York, United States: IBM Corp. Released 2019). 

All the procedures performed in studies involving human participants were in accordance with the 1964 Helsinki declaration (or Declaration of Helsinki) and its later amendments or comparable ethical standards. This study was approved by the Ethics Committee of the University Duisburg-Essen (15-6429-Bo). Informed consent was obtained from all the subjects involved in this study. Data were provided voluntarily by the patients.

## 3. Results

A total of 588 respondents from all over the world were diagnosed with HHT, and 430 of them (73%) answered the questionnaire section about infections and allergies. As not all the questions were completed in full, all the numbers presented in the results are given in relation to sufficiently answered questions. Most patients lived in North America (n = 210/304, 69%) or Europe (n = 60/304, 20%) and had a mean age of 55 years (±SD: 12 years, minimum (min)–maximum (max): 20–83 years). A total of 295 of the 430 patients were female (69%), and most patients reported an endoglin mutation. Regarding the Curaçao criteria, 185 of the 418 patients with HHT were diagnosed with PAVM (44%), and 416 patients suffered from recurrent nosebleeds (epistaxis (n = 411/429, 96%; ESS: 5.8 ± 2.2) and/or gastrointestinal bleedings (n = 148/409, 36%), Table 1). Consequently, 309 of 386 patients were found to be anemic (80%; hemoglobin level: 10.8 ± 2.6 g/dL), with the majority taking iron supplementation and/or receiving blood transfusions (iron supplementation: n = 358/429, 83%; blood transfusions: 119/318, 37%). 

**Figure 1:**
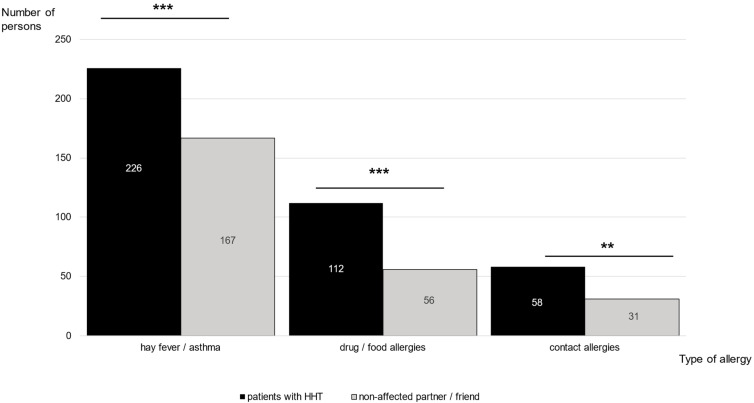
Allergies in patients with HHT and their non-affected partner or friend. The question “Do you have allergies?” was answered by 410 patients with HHT, out of which 276 patients stated an allergy (67%). Meanwhile, 393 patients responded to the question “Do your partner/friend of the same age have allergies” and showed that 50% suffered from an allergy. Patients with HHT and the non-affected controls were compared on the number and type of allergies reported (hay fever/asthma: *p* < 0.001; drug / food allergies: *p* < 0.001; contact allergies: *p* = 0.004; see also Table 2). Patients with HHT suffered significantly more often from allergies than their partner or friend (*p* < 0.001), mostly from hay fever and asthma (type I allergies). *p* = *p*-value, ** *p* ≤ 0.01, *** = *p* ≤ 0.001. Data are shown in number of patients (n) and percentage (%). To compare patients with HHT with the non-affected controls, McNemar test was used. Multiple answers were possible.

To compare patients with HHT with healthy controls, the respondents were asked to compare themselves with their non-affected partners or friends. Only one female patient stated that her male partner also suffered from HHT. Most partners or friends were of the opposite sex (number of male patients/female partner or friend: 111/117, 95%; number of female patients/male partner or friend: 221/224, 99%) and of a similar age (1 ± 7 years younger or older than their affected partner, *t*-test: *p* = 0.16). Compared to the non-affected controls, the patients with HHT had twice the risk of developing an allergy (Table 2), with most patients reporting type I allergies (e.g., hay fever, asthma; Figure 1). 

Clinical factors, such as bleeding parameters or nasal treatment, did not show any significant association with the different types of allergies reported in patients with HHT (Table 3). 

Patients with HHT were at a higher risk of suffering from a sinusitis compared to the non-affected controls (Table 2). Patients using nasal self-packing (regardless of the products used by the patients (medical or non-medical, such as tissue, OR: 0.98, 95%CI: 0.54–1.78)) reported a more frequent history of sinusitis but also showed a higher risk for dermal and wound infections. Patients with PAVM suffered more often from pulmonary infections than the non-affected controls (Table 3). A total of 204 of the 395 patients with HHT stated the need for frequent physician visits (52%) and 121/399 reported hospitalization (30%), which was more frequent than the non-affected controls. In general, the frequency of infections did not differ significantly between older and younger patient/control pairs (*p* > 0.05). 

**Figure 2:**
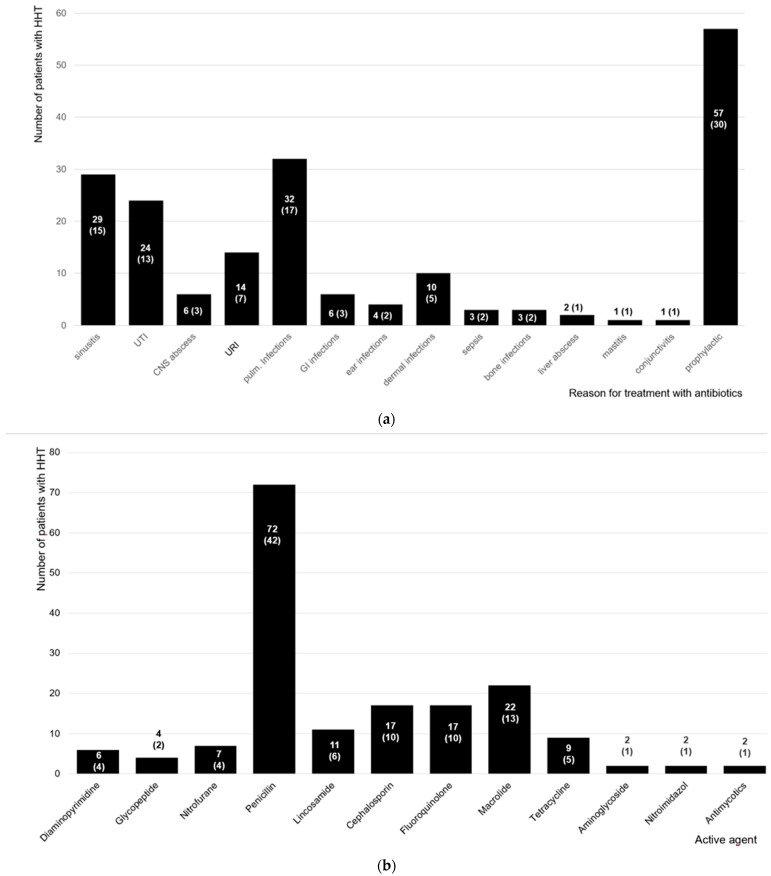
Antibiotic treatment in patients with HHT. (**a**) Reason for treatment with antibiotics in patients with HHT. A total of 152 patients with HHT stated the reason for having taken antibiotics (sometimes multiple reasons were provided, with a total of 192 reasons reported). Most patients suffered from sinusitis, urinary tract infections (UTI), and pulmonary infections. More than one third took an antibiotic prophylaxis prior to dental or surgical procedures. (**b**) Treatment in patients with HHT. A total of 120 patients with HHT documented the prescribed medication (multiple answers were possible; in total, 171 active agents were reported). Most patients were treated with Penicillin, Macrolides, Fluoroquinolones, and Cephalosporins. Two patients reported an antimycotic treatment. CNS = central nervous system; GI = gastrointestinal; and URI = upper respiratory tract infection. Data are presented in terms of the number of patients and the percentage.

Regarding antibiotic intake, 152 of 402 patients had been prescribed antibiotics (38%) more frequently than the non-affected controls. More patients with a PAVM diagnosis than those without stated that they needed more antibiotics than the non-affected controls (OR: 2.26, 95%CI: 1.42–3.60). However, 51% of patients with PAVM did not report prophylactic antibiotic intake (n = 40/79). Although no PAVMs had been diagnosed, 12 of the 121 patients without pulmonary shunts stated that they took an antibiotic prophylaxis (10%). Penicillin was the active agent prescribed most frequently (n = 72/171, 42%; Figure 2). A total of 16 days was the average duration when antibiotic intake was described for therapy (±SD: 41 days, median: 7 days, min: 1 day, max: 365 days, n = 115). 

**Figure 3:**
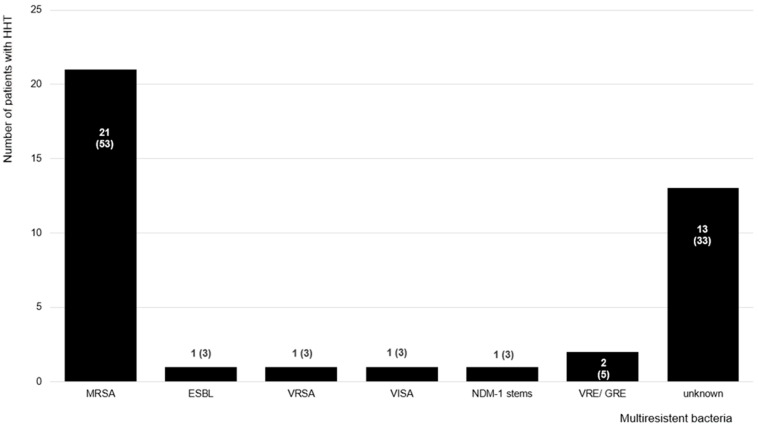
Multi-resistant bacteria in patients with HHT. A total of 40 of the 292 patients with HHT stated that they were diagnosed with multi-resistant bacteria (14%). More than half of these were MRSA. Compared to the non-affected controls, patients with HHT had contact with multi-resistant bacteria more often (McNemar test: *p* = 0.002, OR: 0.36, 95%CI: 0.15–0.69). MRSA = methicillin-resistant *staphylococcus aureus*; ESBL = extended-spectrum β-lactamase-producing germs; VRSA = vancomycin-resistant *staphylococcus aureus*; VISA = vancomycin intermediary sensible *staphylococcus aureus*; NDM-1 = New Delhi metallo-β-lactamase-1; VRE/GRE = vancomycin/glycopeptide-resistant *enterococcus*. Data are presented in terms of the numbers of patients and the percentages; multiple answers were possible, and, in 13 patients, the exact bacteria were not mentioned.

Over ten percent of patients were diagnosed with multi-resistant bacteria, especially methicillin-resistant *staphylococcus aureus* (MRSA; Figure 3). Patients were equally affected, regardless of sex or origin (x^2^ test: p = 0.17; and 0.87, respectively). Compared to the non-affected controls, patients with HHT were not at a higher risk of having been diagnosed with multi-resistant bacteria (McNemar test: *p* = 0.002; OR: 0.36, 95%CI: 0.15–0.69). However, patients who had a higher hospitalization rate and those who suffered from epistaxis were at a higher risk of colonization or infection with multi-resistant bacteria (hospitalization rate: OR: 3.95, 95%CI: 1.89–8.25; epistaxis: OR: 2.18, 95%CI: 0.27–17.36). Patients with HHT who applied nasal self-packings reported more contact to multi-resistant bacteria than those without nasal packings (OR: 4.22, 95%CI: 1.44–12.36). Of these, there was no difference between the patients who applied medical nasal packings and those who used non-medical nasal packings (e.g., tissue; OR: 0.47, 95%CI: 0.22–1.01). 

## 4. Discussion

The study presented here contains patients with HHT from different continents answering an online questionnaire in English or German about allergies and infections. To our knowledge, this is the first report to not only analyze allergies in HHT but also show that patients with HHT suffered significantly more often from allergies than the non-affected controls. Type I allergies such as hay fever were particularly more frequent. In type I and II allergies, immunoglobulins (e.g., Ig E and Ig M) are important mediators. Other studies reported altered levels of immunoglobulins in patients with HHT compared to healthy individuals (increased levels of immunoglobulin A and G and lower levels of immunoglobulin M in patients with HHT) [7,14]. One can speculate that these differences may play a role in patients’ predisposition to allergies. However, further conclusions cannot be made based on the two questions regarding allergies in our questionnaire. 

Based on our questionnaire, patients with HHT reported type IV allergies more often than the non-affected controls. In other experimental studies, it was shown that memory T helper cells play an important role in type IV reactions, such as contact allergies, and a lymphopenia with decreased T and B cells releasing different immunoglobulins in patients with HHT was detected [7,14]. These observed alterations in different types of immunoglobulins, T and B cells may also contribute to the high prevalence of allergies in patients with HHT. Our questions were limited to simple examples like hay fever, asthma, contact, drug, and food allergies so that it would possible to answer them without the need for medical expertise. For this reason, our questions did not cover all types of allergies. Further studies, particularly regarding immunoglobulin E (playing an important role in allergy type I) or reactions to blood transfusions, will be needed to understand the elevated predisposition of patients with HHT to allergies. 

The occurrence of sinusitis was reported more often by patients with HHT compared to the non-affected controls. Patients who applied nasal self-packings reported a noticeably higher rate of sinusitis and wound infections than those without. Possible reasons for this might be recurrent manipulations of the nose and blockage of the ventilation of paranasal sinuses by packings due to epistaxis and/or a higher susceptibility for bacterial infections, as reported in the literature for patients with HHT [19]. It is suggested that nasal colonization of *Staphylococcus aureus* and frequent, prolonged epistaxis might generate a gateway for the hematogenous dissemination of bacteria [20,21]. Nasal packing for treating recurrent epistaxis may lead to infections of the nasal mucosa, which can result in a toxic shock syndrome [22,23]. An increased incidence of bacteremia in patients who used nasal packings to treat recurrent epistaxis was published [24,25]. It is important to mention that other studies have reported an inaccurate interpretation of reported nasal symptoms being misdiagnosed as recurrent acute sinusitis [26]. This could mainly lead to false-positive results. Unfortunately, our study design did not allow for the evaluation of potential correlations between epistaxis and bacterial colonization. However, in our cohort, patients who received nasal surgery (laser treatment, coagulation, septodermoplasty, and nasal closure) were at a higher risk of wound infections than self-reported sinusitis.

Metanalyses of sepsis in the normal population of the United States [27] and Europe [28] showed an incidence of <1–240 in 100.000. In this study, 5% of the patients with HHT were more frequently diagnosed with sepsis than the non-affected controls. Lenato and colleagues published the theory of an age-related immunodeficiency in patients with HHT [9]. However, as our cases and controls are age-matched, the theory of Lenato et al. is not sufficient to explain the difference observed in our study. Additionally, by comparing younger with older patient/control pairs, we were not able to observe hints for an age-dependent effect. Previous studies have noticed changes in the innate and adaptive immune system in patients with HHT. Genetic mutations in patients with HHT occur in the TGF-β pathway, which regulates myeloid cell activation. Therefore, patients’ innate and adaptive immune responses could be affected [29]. In agreement with this theory, alterations in the cells of the immune system (neutrophils, monocytes, and T cells) in patients with HHT have been detected [7,8,10,19].

In addition, we succeeded in demonstrating that patients with HHT who required iron supplementations reported sepsis and abscesses more frequently than those without an iron treatment. It is widely accepted both that micronutrients such as iron are essential for metabolic processes and biochemical activities in cells [30] and that iron plays an important role in B and T cell proliferation in the immune system. Variations in the iron status may affect the immune response in patients with HHT, and those with iron deficiency may have impaired antibody response and immune defense [31,32]. Furthermore, iron supplementation may also affect patients’ immune cells and the growth and virulence of microbial pathogens [33]. However, there was a broad range of 95% confidence intervals in patients reporting sepsis regardless of whether they took iron supplements. Other parameters related to bleeding did not noticeably affect the infection frequencies investigated in our study. Additionally, we could not observe any significant correlations between iron substitution and the prevalence of infections, except for sepsis. 

In our cohort, most patients suffered from HHT type 1, and 44% of the patients with HHT were diagnosed with pulmonary shunting, which is associated with a reduced pulmonary filter function which can lead to abscesses of the brain and other organs. This is likely also the main reason for the higher rate of abscesses of the central nervous system and liver abscesses found in patients with HHT compared to the non-affected controls (see Table 2 and Table 3). The international guidelines recommend an antibiotic prophylaxis in patients with PAVM. In line with the recommendations, most patients took Penicillin [34,35], which is also a treatment option for the infections typically found in HHT cohorts [36]. Interestingly, 10% of patients without PAVM also reported prophylactic antibiotic use, which is not in line with the international guidelines. This suggests that there is a need to intensify awareness campaigns to distribute the correct guideline content among physicians and patients with HHT. 

As found in other studies [20,37], 52% of patients with HHT reported an elevated frequency of medical consultations compared to their healthy partners or friends. Interestingly, only 30% of patients with HHT reported a higher hospitalization rate compared to the non-affected controls. Healthcare-acquired infections are important adverse events in patients with chronic diseases like HHT and are also responsible for longer hospital stays, more frequent and longer antibiotic intake, and increased healthcare costs [38,39]. Hospitalized HHT patients were at a higher risk of being colonized or suffer from infections with multi-resistant bacteria such as MRSA. However, the precise reasons for every hospitalization and the localization of the colonization or infection with multi-resistant bacteria were not documented in this study. Although the prevalence of multi-resistant bacteria may differ from country to country [40], we could not observe any significant differences in patients with HHT from different origins. In our cohort, most patients with HHT came from North America and Europe, with a prevalence of MRSA contact of 0.07%. In another study comparing data through national surveillance systems from different European countries between 1999 and 2002, the prevalence of MRSA varied between 0.04% and 40% [40]. 

### Limitations

The methodological limitations of survey-based online studies are widely known when compared with data acquired by consultations and examinations. In this study, the data about infections and allergies were based on patients’ answers, and the allergies were self-defined. No further information regarding skin prick tests, epicutaneous skin tests, or respiratory function tests was available. However, a high quantity of data from patients from different countries was provided. In order to receive accurate data, we advised the survey respondents to check the data in their medical records, if possible. As the online questionnaire was published in two different languages, patients coming from German- and English-speaking regions were probably over-represented. Interestingly, considering that HTT is an autosomal dominant trait, in our cohort, more female than male patients with HHT answered the survey. However, we could not observe an influence of patient gender on most types of infections. Patients suffering from PAVM and cerebral vascular malformations were represented in our study in a higher proportion than what is usually reported in the literature (PAVM: 44% vs. around 30% [41]; cerebral vascular malformations: 13% vs. 10% [42], respectively). This is possibly due to the fact that, in our cohort, most patients suffered from HHT type 1. The descriptions of the frequency of gastrointestinal bleeding [43] and hepatic vascular malformations [44] were comparable to those of other studies. Patients’ data were compared to their non-affected partners or friends of the opposite sex but the same age and with an assumed, similar social status.

## 5. Conclusions

To the best of our knowledge, our study is the first to indicate that patients with HHT have an increased predisposition to allergies such as hay fever and asthma and drug and food allergies. We also observed a higher risk for sinusitis, urinary tract infections, abscesses of the central nervous system, pulmonary infections, and liver abscesses in patients with HHT. Following our findings, this article recommends that future studies seek further clarification by studying type I and II allergies. Low iron levels requiring iron supplementation can compromise the immune system and may facilitate hematogenous bacterial dissemination, requiring a frequent and long intake of antibiotics. These mechanisms probably contributed to the higher infection and hospitalization rates in the patients with HHT in our study. The prevention of iron deficiency anemia can reduce these consequences, thereby reducing healthcare costs and improving patient care. In patients with HHT with PAVM or in whom PAVM has not been excluded, antibiotic prophylaxis prior to dental or surgical procedures with bacteremia is an important pillar of clinical care. The data in our study suggest that this recommendation is not sufficiently known, as the patients without PAVM also received such a prophylaxis. Therefore, an intensified awareness campaign seems to be a sensible and logical step forward.

## Figures and Tables

**Table 1:** Clinical manifestations of patients with HHT.

			All Patients (n = 430)	Female (n = 295)	Male (n = 135)
Family history		yes	402 (94)	281 (95)	121 (90)
		no	17 (4)	7 (2)	10 (7)
		n.k.	11 (3)	7 (2)	4 (3)
		mv	0	0	0
Epistaxis		yes	411 (96)	279 (95)	132 (96)
		no	18 (4)	16 (5)	2 (2)
		mv	1 (0.2)	0	1 (2)
TAE		yes	412 (96)	279 (95)	133 (99)
		no	12 (3)	10 (3)	2 (2)
		n.k.	6 (1)	6 (2)	0
		mv	0	0	0
Visceral lesions	GI	yes	148 (36)	97 (34)	51 (40)
		no	121 (30)	80 (28)	41 (32)
		n.k.	140 (34)	105 (37)	35 (28)
		mv	21	13	8
	PAVMs	yes	185 (44)	131 (45)	54 (42)
		no	153 (37)	101 (35)	52 (41)
		n.k.	80 (19)	58 (20)	22 (17)
		mv	12	5	7
	HVM	yes	96 (24)	72 (26)	24 (19)
		no	153 (38)	94 (34)	59 (47)
		n.k.	157 (39)	114 (41)	43 (34)
		mv	24	15	9
	CVM	yes	55 (13)	38 (13)	17 (13)
		no	247 (60)	168 (59)	79 (62)
		n.k.	108 (26)	77 (27)	31 (24)
		mv	20	12	8
			**All Patients** **(n = 175)**	**Female** **(n = 124)**	**Male** **(n = 51)**
Genetics	HHT Type 1		65 (37)	50 (40)	15 (29)
	HHT Type 2		106 (61)	72 (58)	34 (67)
	SMAD 4		3 (2)	1 (1)	2 (4)
	HHT Type 5		1 (0.2)	1 (1)	0

Most patients with HHT suffered from telangiectasia and epistaxis (96%, respectively). A total of 303 patients reported that visceral malformations had been diagnosed (n = 303/425, 71%). In 175 patients (n = 175/430, 41%), the result of genetic testing was reported. Data are shown in number of patients (n) and percentage. TAE = telangiectasia; GI = gastrointestinal involvement; PAVMs = pulmonary arteriovenous malformations; HVM = hepatic vascular malformation; CVM = cerebral vascular malformation; n.k. = not known; and mv = missing value.

**Table 2:** Comparison of different infections and allergies in patients with HHT and their non-affected partner or friend.

	Patients with HHT n (%)	Non-Affected Partner/Friend n (%)	Odds	95% Confidence Interval	*p*-Value ^1^
Infections					
Sinusitis	152 (40)	60 (16)	2.53	1.89–3.55	0.001
UTI	119 (32)	56 (15)	2.13	1.54–3.03	0.001
CNS abscess	18 (5)	5 (1)	3.61	1.29–12.40	0.011
IUA	94 (25)	91 (24)	1.03	0.76–1.41	0.883
Pulm. infections	66 (18)	32 (9)	2.06	1.35–3.33	0.001
GI infections	75 (20)	51 (14)	1.47	1.02–2.17	0.040
Dermal infections	38 (10)	23 (6)	1.65	0.97–3.03	0.072
Sepsis	19 (5)	15 (4)	1.26	0.60–2.86	0.607
Bone infections	26 (7)	28 (8)	0.93	0.51–1.67	0.892
Liver abscess	30 (8)	2 (1)	15	3.81–129.54	0.001
Wound infections	41 (11)	32 (9)	1.28	0.79–2.12	0.349
Allergies					
Any allergy	112 (43)	43 (22)	2.61	1.85–3.91	0.001
Hay fever/asthma	100 (46)	47 (29)	2.13	1.51–3.15	0.001
Drug/food allergies	82 (77)	31 (56)	2.65	1.77–4.33	0.001
Contact allergies	41 (79)	18 (62)	2.28	1.32–4.58	0.004

In this table, we have documented the answers of patients with HHT who reported more (column 1)/less (column 2) infections and allergies compared to the non-affected controls. Patients with HHT suffered significantly more often from sinusitis, UTI, CNS abscesses, pulm. Infections, and liver abscesses and had a higher risk of developing allergies than their non-affected partner or friend. Patients who were equally affected or patients who stated that neither they nor their non-affected controls suffered from the above-mentioned infections/allergies are not shown. The number of patients who answered these questions varied between 367 and 376. ^1^ McNemar; HHT = hereditary hemorrhagic telangiectasia; n = number of patients; % = percentage; UTI = urinary tract infections; CNS = central nervous system (numbers too small for calculating odds ratio and 95%CI); IUA = infections of the upper airway; and GI = gastrointestinal. Multiple answers were possible when reporting allergies.

**Table 3:** The analysis of different bleeding indicators, nasal treatment options, and the presence of PAVMs regarding different types of infections and allergies.

	Anemia	ESS	Iron Supp.	Blood Transfusion	Nasal Surgery	Nasal Packing	PAVMs
Sinusitis	0.27 0.09–0.79	1.43 0.64–3.20	0.42 0.15–1.15	1.24 0.58–2.64	1.28 0.69–2.39	4.95 2.85–8.59	1.41 0.72–2.75
UTI	0.85 0.36–2.01	0.54 0.22–1.37	0.84 0.36–1.98	0.75 0.33–1.71	0.69 0.35–1.38	0.31 0.162–0.6	1.12 0.54–2.32
CNS abscess	-	-	0.65 0.06–7.32	1.25 0.09–17.65	1.73 0.22–13.67	3.33 0.38–29.39	1.88 0.13–26.32
Other abscesses	-	2.33 0.74–7.35	9.00 0.44–183.97	-	-	3.14 0.17–57.08	-
IUA	1.16 0.55–2.46	0.80 0.37–1.75	0.88 0.39–1.97	1.16 0.55–2.49	1.00 0.54–1.87	0.85 0.45–1.62	0.95 0.50–1.83
Pulm. infections	0.89 0.28–2.85	0.87 0.29–2.62	0.8 0.23–2.78	1.49 0.54–4.08	1.28 0.51–3.25	1.19 0.44–3.22	8.84 3.69–21.20
GI infections	0.82 0.32–2.10	0.78 0.27–2.24	0.37 0.13–1.08	2.98 0.99–8.99	1.43 0.66–3.11	1.31 0.57–3.02	1.01 0.41–2.45
Dermal infections	2.78 0.59–13.04	2.00 0.47–8.49	-	1.46 0.36–5.91	0.83 0.26–2.68	5.30 1.40–20.12	1.46 0.45–4.66
Sepsis	0.46 0.04–4.98	-	9.00 0.92–88.16	2.5 0.37–16.89	0.4 0.07–2.45	2.13 0.31–14.73	0.4 0.08–2.12
Bone infections	0.40 0.03–4,74	1.50 0.21–10.81	0.66 0.13–3.28	1.27 0.33–4.98	1.14 0.35–3.78	0.69 0.20–2.46	0.66 0.19–2.31
Wound infections	1.36 0.31–5.97	0.53 0.11–2.43	0.96 0.20–4.62	1.77 0.61–5.17	1.58 0.58–4.29	4.53 1.29–16.25	2.73 0.94–7.89
Allergies	1.34 0.77–2.35	0.68 0.38–1.21	1.22 0.70–2.11	1.14 0.69–1.87	1.05 0.68–1.64	0.92 0.57–1.48	1.31 0.82–2.07
Hay fever/asthma	0.618 0.37–1.04	1.03 0.61–1.74	1.03 0.61–1.75	0.94 0.59–1.50	1.39 0.92–2.10	1.01 0.65–1.58	1.23 0.80–1.91
Drug/food allergies	0.98 0.55–1.71	0.72 0.41–1.26	1.48 0.78–2.79	0.88 0.53–1.48	1.11 0.70–1.76	1.17 0.70–1.93	1.61 0.98–2.66
Contact allergies	1.10 0.52–2.30	0.63 0.29–1.37	1.05 0.49–2.26	0.96 0.47–1.94	0.81 0.45–1.46	1.80 0.87–3.71	1.33 0.69–2.56

Patients who used nasal packings more often reported dermal infections, wound infections, and sinusitis compared to those without nasal packings. Patients with pulmonary arteriovenous malformations (PAVMs) suffered more often from pulmonary infections compared to those without PAVMs. The odds ratios (number in upper line) with 95% confidence intervals (numbers in lower line) are shown, calculated for patients with HHT who suffered more or less often from different infections compared to the non-affected controls (‘-’ = numbers were too few to analyze). Nasal surgery included, for example, laser treatment, coagulation, septodermoplasty, and nasal closure; iron supp. = iron supplementation (oral and/or intravenous), ESS: score < 5 vs. score ≥ 5; UTI = urinary tract infections; CNS = central nervous system; and GI = gastrointestinal. The number of patients who answered these questions varied between 213 and 410.

## Data Availability

The datasets used and/or analyzed during the current study are available from the corresponding author upon reasonable request.

## Supplementary Materials

The following supporting information can be downloaded at https://www.mdpi.com/article/10.3390/jcm13133752/s1, Supplement material S1: Stratification of diagnostic assignments; Supplement material S2: Survey about Hereditary Haemorrhagic Telangiectasia (HHT) (also known as Osler-Weber-Rendu Syndrome).
