# Supplementary material for: Increased Risk for Infections and Allergic Disease in Hereditary Hemorrhagic Telangiectasia
Source: J Clin Med. 2024 Jun 27;13(13):3752. doi: 10.3390/jcm13133752 (PMC11242906; doi:10.3390/jcm13133752)
Supplement: Supplementary file 1 [file jcm-13-03752-s001.zip › Revision_supplement material 2_questionnaire.pdf]

Survey about Hereditary Haemorrhagic Telangiectasia (HHT)
(also known as Osler-Weber-Rendu Syndrome)

Conducted by the University of Essen in cooperation with the German HHT patient association "Morbus Osler Selbsthilfe e.V."

Dear HHT patient,

this study about the disease hereditary hemorrhagic telangiectasia received a positive voting by the university Duisburg-Essen Ethics Commission and is operated by the University of Duisburg-Essen ENT (ear nose throat) clinic in Essen (Trial researchers are Prof. Dr. med. U. Geisthoff and Dr. med F. Droege, you will find their contact data below) in cooperation with the Morbus Osler Selbsthilfe e.V. (HHT patient association Germany). Typical signs of the disease associated with HHT are: multiple telangiectases (vascular enlargements) and vascular malformation (improperly grown vessels), especially present on mucous skin (mucosa), and vessels in the gastro intestinal tract, liver, brain and lungs.
Recurrent bleedings, especially frequent nose bleeds are characteristic signs of the disease. We would like to improve the detection and treatment of the disease HHT by gaining new insights and knowledge. We invite you cordially to tell us your experiences by answering the questions in this survey. The survey covers 6 main parts (general disease history, self tampon insertion, questions about feelings of guiltiness, questions about the restless leg syndrome, hormonal influence of nose bleeds, and questions about your susceptibility for infection). This survey will take about 30 minutes, on the average. The results are of a great importance for the research about the disease HHT (Rendu Osler Weber's disease).
You will be asked about data protection regarding your personal data. The company involved in this survey offers high security standards for data transmission (see also the company's privacy policy). The data will be stored transitionally on the online provider's server (of surveymonkey.com), before it will be transferred via secure data transmission to the studying physicians. In the past, studies about HHT have been performed using this survey company, without any problems regarding data security. However, we still have to make you aware that 100% security of electronic communication on the internet cannot generally be warranted. Participation in the survey will probably be conducted until May 2017; afterwards we will analyze the collected data. Participants must be of full legal age (grown up). The participation is voluntary. Non-participation has no adverse consequences on your treatment. The participation in the survey bears no additional risks for you. You can refuse your participation in this trial at any time, without explanation. In case that you should want to withdraw your participation at a later date, we can only delete your data, provided you give us your e-mail-address as this would be the only way to identify your data. Anonymous or previously published data cannot be deleted.

Our goal is to analyze your answers and to use information to gain new findings to optimize the detection and therapy of HHT (hereditary hemorrhagic telangiectasia, also known as morbus Rendu Osler Weber).

Guidance for filling in the survey: If you click on the button "next" or "back" you will be forwarded to the next or last question and can be used for re-editing your answers. If you click on words marked in blue, this leads you to a webpage with additional information about the disease HHT and definitions.
Please be aware, that we cannot take any responsibility for the content of any external pages which might be linked to this survey. Take as much time as you need to answer the survey, several hours or days, including time out to read other related medical reports or findings if necessary. If you have to interrupt your work while taking the survey, just make sure the webpage remains open; otherwise all data will be lost. After the last question, please click on "ready" to end the survey. Immediately after clicking "ready", your data will be transmitted to the physicians participating in this study - Prof. Dr. med. U. Geisthoff and Dr. med. F. Droege. After clicking the finish button, the answers cannot be changed. The website shouldn't be closed while answering the questions because this would lead to data loss and you will have to start the survey once again from the beginning.
It is possible that multiple persons can take part in the survey, but this has to be done in sequence, one person at a time. It is not possible to work parallel on two surveys on the same computer. The next person can start with the survey after the predecessor has finished the survey and sent it for completion.
Please answer the questions with complete answers when possible. Some questions might require that you have your medical history alongside for reference, or doctor's reports/various medical documents, so that your answers reflect your medical history in answering the questions. The questions are straightforward, but it could still be possible that you don't understand the entire question content immediately. For example: there are a few questions which you should only answer once, as double entries by one person should be avoided. (See question 2 below). If you don't want to answer a question, simply leave the fields open (exceptions are the fields marked with '*', which must be answered. Note: only by answering these questions with * you can access the next question). If you should have relatives who cannot fill out the survey for themselves, but who would accept your filling out the survey for them, we would be very thankful for your assistance in helping them. Many thanks for your support which will enable us to gain a better understanding of HHT.

With best regards,

Professor Dr. med. U. Geisthoff and Dr. med. F. Droege

Clinic for ENT-medical science of the University Clinical Center Essen, Hufelandstraße 55, D-45147 Essen, Germany, Tel.: 0049-201-7232386

1. Are you filling out the survey for yourself or a relative?

- ◯ For myself
- ◯ For a relative

*2. Where are you from?

- North Africa
- South Africa
- Asia
- Southeast Asia
- Middle East
- Australia
- Western Europe
- Eastern Europe
- South America
- North America
- Central America
- Other (please name)

3. It is important that no person is registered twice. Could you please tell us, in which city your parents and grandparents live / lived recently? (If the parents/grandparents live in the same city, please write "#")

**Your mother lives/lived in**	
**Your father lives/lived in**	
**Your father's father lives/lived in**	
**Your father's mother lives/lived in**	
**Your mother's father lives/lived in**	
**Your mother's morther lives/lived in**	

4. Does this disease also exist among your relatives?

- ○ Yes
- ○ No
- ○ I don't know

5. If yes, who is affected (please keep in mind, that normally only one parent or grandparent can be affected)? (Multiple selections are possible)

- ☐ Father
- ☐ Mother
- ☐ Brother(s) or sister(s) (Please check with a cross if at least one brother or one sister has HHT.)
- ☐ Children (Please check with a cross, if at least one child has HHT.)
- ☐ Grandchildren (Please check with a cross, if at least one grandchild has HHT.)
- ☐ Grandfather mother's side
- ☐ Grandmother mother's side
- ☐ Grandfather father's side
- ☐ Grandmother father's side
- ☐ Cousin(s) (please check with a cross if at least one cousin has HHT)

Other relatives (please list below)

____

6.

Please tell us how old (age in years) the following relatives were when they passed away, and what the cause of death was. For example: Grandfather mother's side: 85 years, died from heart attack, or one of 3 children died 15 years old from lung bleeding (hemoptysis). (If your relatives are still alive, please leave the field empty.)

**Father**	
**Mother**	
**Brother(s) or sister(s)**	
**Children**	
**Grandfather mother's side**	
**Grandmother mother's side**	
**Grandfather father's side**	
**Grandmother father's side**	

Your disease history/ diagnosis:

* 7. Have you been diagnosed with HHT / Osler-Weber-Rendu Syndrome?

- ◯ Yes
- ◯ No

8. When was the disease diagnosed? (Please enter the approximate date / year)

[ ]

9. Who diagnosed the disease?

- ◯ ENT-physician
- ◯ Family physician (primary care physician)
- ◯ Internist (Cardiologist) (= physician specialized in heart and circulation) / Pulmonologist/Pulmonary Specialist (= lungs specialist) / Gastroenterologist (=specialized in stomach and intestine diseases))
- ◯ Hematologist (= specialist for blood disorders, like anemia)
- ◯ Others (please name)

10. What were your first symptoms/conditions?

[ ]

11.
How old were you when you noticed or experienced the first symptoms/ conditions (for example: Nose bleeds or visible vascular changes or other consequences associated with HHT, i.e. such as a stroke? (Age in years)

[ ]

12. Do you have the feeling that the course of your disease in general

- ◯ Worsened over time
- ◯ Remained stable constant)
- ◯ Improved over time?

13. Has your HHT been confirmed genetically (blood work for genetic testing)?

[ ]

14. If yes, do you know, which genetic type (genotype) was found?

- ○ HHT1 (Endoglin)
- ○ HHT2 (Alk1 or ACVRL1)
- ○ others (please name)

Your disease laboratory history values (blood parameters):

15. How high has your hemoglobin value (Hb, red blood color) been on average during the past 2 years? (mg/dl)

16. Have you ever had to receive blood transfusions?

- Yes
- No

17. If yes, how many blood transfusions have you ever received in your life (approximately)?

18. Do you have more/frequent/stronger bleedings - dependent on your hemoglobin value (Hb-value)?

- No
- Yes, when the Hb value is higher
- Yes, when the Hb value is lower

19. Have you ever used iron supplements/ preparations?

- Yes
- No

20. If yes, in which form (multiple answers possible)

- Pills or drops
- Infusions (for example - iron Infusion)
- Others (please name)

Your disease history with telangiectasia(s):

(Telangiectasia = small vascular enlargements, as red spots, especially in the regions of mouth, lips, face and fingers)

21. Do you have the typical telangiectasia(s)?

- ◯ Yes
- ◯ No
- ◯ I don't know

22. If yes, how old were you when you became aware of the appearance of the firstelangiectasia (Age in years)

[ ]

23. Is the number of telangiectasia

- ◯ growing
- ◯ constant
- ◯ getting less?

Your disease history involving inner organs:

24. Organs:

	Yes	No	I don't know
Brain	☐	☐	☐
Lungs	☐	☐	☐
If yes: Are the lungs affected in all areas, or are there areas which are not treatable?	☐	☐	☐
Liver	☐	☐	☐
If yes: Do you have a liver areas requiring treatment, so that you receive medication or have had an embolization or transplantation	☐	☐	☐
Stomach and intestines (guts)	☐	☐	☐

Others (please list below)

[ ]

25. Which of the following examinations have you had:

	Yes	No	I don't know
Computed tomography (CT scan) / Magnet resonance imaging (MRI) of the brain	◯	◯	◯
Echocardiogram /heart echo/ heart ultrasound with contrast agent to evaluate cardiac power (cardiac output) and indirect examination of the lungs	◯	◯	◯
Computed tomography (CT/Cat Scan)/Magnetic Resonance Imaging (MRI) of the lungs	◯	◯	◯
Ultrasound (sonography) / Computed Tomography (CT) / Magnetic Resonance Imaging (MRI) of the liver	◯	◯	◯
Gastroscopy (imaging of the stomach) Esophago (esophagus) gastro (stomach) duodenscopy (EGD), Swallowed a Video camera pill, Colonoscopy (large intestine)	◯	◯	◯

In case there were examinations you have experienced which are not mentioned above, please indicate them below:

26. Do you know if vascular malformations in the stomach or intestines are bleeding or have bled in the past?

◯ Yes, I have or had gastro intestinal bleeding

◯ No, no I've never had gasto intestinal bleeding

◯ I don't know

27. Have you ever had a suppurative focus (abscess, pus containing bulb) or an infarct/ progressive stroke of the brain, liver, spleen or other organs?

◯ Yes

◯ No

Your disease history - Nose bleeds (= Epistaxis):

28. Do you have nose bleeds?

- ◯ Yes
- ◯ No

29. If yes, how old were you when your nose bleeds began? (Please enter your age in years)

[ ]

30. Are your nose bleeds still being treated?

- ◯ Yes
- ◯ No

31. If yes, how often?

[ ]

32. In relation to the last 4 weeks:
How often do you have nose bleeds, on the average?

- ◯ Less than once a month
- ◯ Once a week
- ◯ Several times a week
- ◯ Once per day
- ◯ Several times a day

33. In relation to the last 4 weeks:
Please estimate the severity of the nose bleeds on a scale from 1 = very slow dropping to 10 = like an open tap

[ ]

34. In relation to the last 4 weeks: How long did the nose bleeds last?

- ◯ <1 minunte
- ◯ 1-5 minutes
- ◯ 6-15 minutes
- ◯ 16-30 minutes
- ◯ >30 minutes

35. How long did your longest nose bleed last?
(please tell in minutes)

36. How many hours per day does the nose bleed and keep you from activities of daily life (time in hours, please)?

37. Have you ever consulted a doctor (physician) because of nose bleeds?

- ◯ Yes
- ◯ No

38. What do you do in case of acute/ heavy nose bleeds?

39. What do you do to prevent nose bleeds (nose care)?

40. Do you use a nosal creme?

- ◯ Yes
- ◯ No

41. Are you taking any pills against recurrent nose bleeds? (For example: Tranxeamic acid = Cyklokapron, Thalidomide, Tamoxifen, ACC, estrogen/ gestagen).

- ◯ Yes
- ◯ No

42. If yes, could you please list the ingredient/medical name (chemical name) below:

43. Has your nose ever been treated with a laser?

- ◯ Yes
- ◯ No

44. If yes,

When was your nose lasered for the first time? (Age in years)

When was your nose lasered for the last time? (Age in years)

How often? (more or less)

45. Did the nose bleeding improve after laser Treatment?

- Yes
- No

46. If you have undergone laser treatment several times, did the intervals between subsequent treatments become shorter?

- Yes, the time intervals between the single treatments have become shorter over time.
  This means that I have the impression that over time,
- No, laser treatments have been applied with the same frequency/intervals of time (same frequency).
- No, the time intervals between the single treatments have become even longer.
  This means, that I have the impression that over time, laser treatment was less often necessary.

47. Have there been other treatments (other than laser) for treating your nose bleeds?

- Yes
- No

48. If yes, what was done?
(for example Septo-Dermo-Plastic, Young's Procedure, Arterial Embolisation procedure, Endoscopic Cauterization) (please check your physician's letters)

Your disease history – nasal self packing:

49. Did you know that during a nosebleed, you can apply a nasal packings in your nose?

- ◯ Yes
- ◯ No

50. Have you ever self treated your nose with nasal packings?

- ◯ Yes
- ◯ No

51. If yes: With what? (multiple choices possible)

- ☐ Tissue handkerchief
- ☐ Haemostypticum (for example Tabotamp®)
- ☐ Styphnasal®
- ☐ Pneumatic low pressure tampon (Rapid Rhino®)
- ☐ Others (please name)

52. Who showed you how to treat your nose by yourself, or respectively, how did you learn?

- ◯ HHT patient association or self aid group
- ◯ Youtube video
- ◯ ENT physician
- ◯ Others (please name)

53. When were you shown the principle of applying nasal packings? (approximate month and year)

54. Do you know other HHT patients who apply nasal packings themselves?

- ◯ Yes
- ◯ No

55. Have you ever encountered complications or difficulties when self-packing your nose?

- ◯ Yes
- ◯ No

56. Have there been complications/difficulties with nasal self packing amongst other persons you know?

- ◯ Yes
- ◯ No

57. If you or others you know have had difficulties or complications during nasal self-packing, could you please describe them below?
(multiple selections are possible; please be aware that it is unknown to us if these very severe complications have ever happened with HHT patients)

- ◯ Swallow nasal packings (in the throat)
- ◯ Hypoxia (lack of oxygen) / choking fit
- ◯ Death
- ◯ Others (please tell)

58. How did you stop nose bleeds before you learned to conduct self packing?

59. How good have you been able to stop nose bleeds by self packing?

- ◯ Absolutely not, it still continued bleeding
- ◯ A little
- ◯ It drops only a little
- ◯ The nose bleeding stopped after a while
- ◯ The bleeding was stopped immediately

60. Before you learned how to apply nasal packings, did you sometimes have the feeling of losing control of the nose bleeding situation?

- ◯ Very often
- ◯ Often
- ◯ Sometimes
- ◯ Rarely
- ◯ Never

61. After you learned how to apply nasal packings, have you had more often/ less often the feeling of losing control of the situation of your nose bleeding?

- ◯ Very often
- ◯ Often
- ◯ No change
- ◯ Less often
- ◯ Never

62. Please compare the time before you learned how to use nasal self packings on your own, with the time after you had learned it.
How has the intake of iron preparations (pills, drops/ injections) and/or blood transfusions changed? (more than one answer is possible)

- ◯ Not at all
- ◯ I have lost less blood after learning to self tampon
- ◯ I have received more iron after self tamponing
- ◯ I needed more blood transfusions after self tamponing

Your disease history – nasal self packing:

If you have ever <u>used nasal packings yourself</u> please answer the following questions:

63. Has the ability to pack your nose affected things you do?

64. Has the ability to pack your nose made your overall life better or worse?

65. Since you learned how to self pack your nose, have you felt more or less optimistic about the future?

66. Since learning how to self pack your nose, do you feel more or less embbarrassed when with a group of People?

67. Since you learned to use nasal self packings, do you have more or less self-confidence?

68. Since you are using nasal packings, have you found it easier or harder to deal with company?

69. Since you have learned to apply nasal self packings, do you feel that you have more or less support from your friends?

70. Have you been to your family doctor, for any reason, more or less often, since you use nasal packings?

71. Since using nasal packings, do you feel more or less confident about job opportunities?

72. Since you learned the ability to use self packings, do you feel more or less self-conscious?

73. Since you use the self packings, are there more or fewer People who really care about you?

74. Since you use nasal packings, do you catch colds or infections more or less often?

75. Have you had to take more or less medicine for any reason, since learning how to pack your nose?

76. Since you use nasal packings, do you feel better or worse about yourself?

77. Since using nasal packings, do you feel that you have more or less support from your family?

- Much more support
- More upport
- No change
- Less support
- Much less support

78. Since learning how to pack your nose, are you more or less inconvenienced by your health problem?

79. Since learning how to use nasal packings, have you been able to participate in more or fewer social activities?

80. Since you are using nasal packings, have you been more or less inclined to withdraw from social situations?

Feelings of guilt:

Many patients report about feeling of guilt in combination with HHT. For this reason, we would like to pick up this theme as well. It could be that addressing this theme might make you angry at yourself, or could cause a stir. If this should be the case and you would like to speak with someone, we, i.e., the Morbus Osler Selbsthilfe e.V. and/or Prof. Dr. Geisthoff and Ms. Dr. Droege are standing by for your support.

81. Did you ever have a feeling of guilt in combination with HHT? (multiple answers are possible)

- ☐ No
- ☐ Yes, because I am a burden to my environment.
- ☐ Yes, because I have passed the disease to my descendants (offspring).
- ☐ Yes, because....(please explain)

82. Do you believe, that you suffer more from feelings of guilt than your partner does?

- ◯ No
- ◯ Yes
- ◯ Equal
- ◯ Not applicable (please explain why)

83. In HHT various other diseases (some kinds of cancer and heart conditions) are less common than those in the general population. Does this knowledge help you or could this knowledge help regarding your feelings of guilt in combination with HHT?

- ◯ Yes, because of this, I feel less guilt.
- ◯ No, it doesn't change anything.
- ◯ It strengthens my feeling of guilt.
- ◯ I don't have feelings of guilt.
- ◯ Other (please explain)

84. In case that you have feelings of guilt in combination with HHT, what could help you to better deal with it?

Restless Leg Syndrome / disease

Restless Legs Syndrome involves an irresistible urge to move certain regions of the human body to stop odd or uncomfortable sensation. This condition often involves the legs, torso, the head or the arms. Moving an affected part of the body modulates sensations, which may provide temporary relief. Questions No. 85 to 99 are about the Restless Legs Sydrome. Please answer these questions as it is unclear if there is a link between HHT and this Syndrome.

85. Have you been diagnosed with Restless Legs Syndrome? (If you click onto the link below, you will find an exact description of the disease.)

- ◯ Yes
- ◯ No
- ◯ I am not sure

86. If yes, were you / are you treated with a medication therapy?

- ◯ Yes
- ◯ No
- ◯ I am not sure

87. If yes, which medication? (please list the name of the drug)

[ ]

88. Are you taking Dopamine / Domaminagonists (like for example: Adartrel, Benserazid, Leganto, Levodopa, Neuropro, Proninorol, Sifrol) in form of a patch or pill?

- ◯ Yes
- ◯ No

89. If yes, which one?

[ ]

90. Do you feel a need for movement of the legs /arms?

- ◯ Regularly (more than 4 from 7 days per week)
- ◯ Occasionally (1.-. 4 days per week)
- ◯ Not at the present

91. Do you feel that when you feel the need for movement, discomfort (paresthesia) of the legs/arms (like pins and needles, prickling, dragging, and pain) occurs?

- Regularly (more than 4 from 7 days per week)
- Occasionally (1.-. 4 days per week)
- Not at the present

92. Does your need for movements begin/ increase/worsen (paresthesia) when you are resting (lying down or sitting?)

- Regularly (more than 4 from 7 days per week)
- Occasionally (1.-. 4 days per week)
- Not at the present

93. Does the need for movement / discomfort (paresthesia) disappear partly or completely by movement (for example like running/walking around or stretching)?

- Regularly (more than 4 from 7 days per week)
- Occasionally (1.-. 4 days per week)
- Not at the present

94. Does the need for movement / discomfort (paresthesia) increase in the evening and/or at night?

- Regularly (more than 4 from 7 days per week)
- Occasionally (1.-. 4 days per week)
- Not at the present

95. Do you suffer from a sleeping disorder (like Sleep Apnea/falling asleep or continual sleepiness)?

- Yes
- No
- Questionable/ I don't know

96. Does someone else among your close relatives (parents, children, sisters or brothers) suffer from the need for movement/discomfort (paresthesia)?

- Regularly (more than 4 from 7 days per week)
- Occasionally (1.-. 4 days per week)
- Not at the present

97. Has your need for movement / discomfort (paresthesia) been improved, after the intake of Dopamine / Dopamine products (for example, Adartrel, Benserazid, Leganto, Levodopa, Neuropro, Ropinorol, and Sifrol)?

- ◯ Yes
- ◯ No
- ◯ Questionable
- ◯ Not applicable (I have never taken such a medication)

98. Have you ever been screened for sleeping disorders, (for example, apolysomnography = test while sleeping), and if so, did this test reveal any signs of Restless Legs Syndrome? (see clinic papers)

- ◯ Yes
- ◯ No
- ◯ Questionable/ I don't know
- ◯ Not applicable (I have never had such a test)

99. Need for movement and discomfort (paresthesia) can be signs of so called Restless Legs Syndrome. However, there are also many other possible explanations. Do you know if there might be another cause of need for movement (or paresthesia) that could explain it in your case?

- ☐ Yes
- ☐ No

If yes, which?

[ ]

If you answered one or more of the questions from 85 to 99 with 'Yes', it is recommended that you talk to your family doctor. These questions are used to diagnose Restless Legs Syndrome. If there could be a possibility that you might have Restless Legs Syndrome (this means you have answered the above mentioned questions with “yes”) we recommend you mention this to your family doctor. Your doctor will possibly initiate further examinations.

Hormones / Anti-hormones

***100. Following are some questions related to female hormones. However, if you are a man, please skip these questions and continue to proceed with the survey.**

- ◯ I am female
- ◯ I am male

Hormones / Anti-hormones

101. Do you take or have you ever taken hormone preparations? (contraceptives, 'the pill', a (copper) coil/intrauterine Devise (IUD), intravaginal ring, patch...) or hormones for menopausal conditions (estrogens / gestagens / Tamoxifen/ Raloxifen/aromatase inhibitors / GnRH-analoga)

- Yes
- No

102. If yes, would you tell please tell us the product name below?

103. How do you / did you take the product (multiple answers possible)

- As a pill
- As a patch
- As a creme
- Nothing applies, because I've never taken any hormone preparations.
- Others (please tell)

104. Why do you take / did you take hormones? (multiple answers possible)

- [ ] Contraception
- [ ] Heavy menstrual period
- [ ] Painful menstrual period
- [ ] Skin and hair problems
- [ ] Irregular cycle
- [ ] Menopausal conditions /discomfort
- [ ] Endometriosis
- [ ] Brest cancer
- [ ] Osteoporosis
- [ ] Nothing applies, I've never taken any hormones

Others (please tell)

105. When did you start taking the preparation? (please indicate the calendar year) (If you have taken more than one product in the past, please enter each time span (in years) when you have taken each product

106. Have you had a surgery in your uterus or ovaries?

- ◯ Yes
- ◯ No

107. If yes, what was done?

- ◯ Uterus (womb) surgery (abdominal hysterectomy)
- ◯ Ovary dissection (single sided)
- ◯ Ovary dissection (both ides)
- ◯ Dissection of an ovarian cyst
- ◯ Other (please tell)

108. What was the reason for the surgery?

109. If yes, when and where did you undergo the surgery?

110. Please indicate how your nose bleeds changed during the following times:

	More	Less	Equal
During puberty	○	○	○
During menstruation	○	○	○
After delivery/ during lactation	○	○	○
Since the above mentioned surgery of ○ the ovaries	○	○	

111. If the matrix above doesn't describe the changes sufficiently, please describe more comprehensively/detailed below: (for example: "Right before menstruation the bleeds used to increase..."

112. Please tell us how the vascular enlargements (telangiectasias) have changed during the following times:

	More	Less	Equal
During puberty	○	○	○
During menstruation	○	○	○
After delivery/ during lactation	○	○	○
Since the above mentioned surgery of ○ the ovaries	○	○	

113. If the matrix above doesn't describe the changes sufficiently, please describe more comprehensively/detailed below: (for example;"Right before… the vascular enlargements seem to become more…")

114. If you have ever been pregnant: Did the vascular enlargements (telangiectasias) increase and/or nose bleeds increase during pregnancy:

- ◯ Reduce/ improve
- ◯ Worsen
- ◯ Remained equal
- ◯ Not applicable (I have never been pregnant)

115. If you have been pregnant and noticed changes in the bleedings frequency/strength/duration, in which week (approximately) of your pregnancy did this appear?

[ ]

116. In comparison to other women your age (on the average), is or was your menstrual bleeding (at the time when you were not taking any hormone preparations) weaker?

- ◯ less
- ◯ about as strong
- ◯ stronger
- ◯ not applicable, because I've never had menstrual bleeding

117. The following numbers refer to your menstrual bleeding without the intake of any hormonal preparations: To estimate the strength of bleeding, please tell how many sanitary napkins or tampons you need/needed per day (not including nights):

- ◯ 1.to.2 / day
- ◯ 3.to.4 / day
- ◯ More than 4 per day
- ◯ Not applicable, because I've never had any menstrual bleeding

118. How long does/ did your menstrual bleeding last?

- ◯ 2.to.3 days
- ◯ 4.to.7 days
- ◯ More than 7 days
- ◯ Not applicable, because I've never had any menstrual bleeding

Susceptibility to infection

119. How old is your partner? (age in years)
If you don't have a partner, please leave this field empty.

[ ]

120. Is your partner female or male?

- ○ Male
- ○ Female
- ○ I don't have a partner

121. Has your partner been diagnosed with HHT?

- ○ Yes
- ○ No
- ○ I don't have a partner

The following questions refer to your partner. If you are single at the moment, or if you not have had a partner yet, please refer to friends of the same age, who don't have HHT.

122. Compared to your partner, do you have more or less often:

	More often	Equal	Less often	Neither I nor my Partner suffer from it
Suppurative focuses in the brain / brain abscesses	○	○	○	○
Suppurative focuses/ abscesses outside the brain (for example in the spleen, liver, lungs, other organs)	○	○	○	○
Gastro-intestinal-infections (stomach or guts)	○	○	○	○
Cold	○	○	○	○

	More often	Equal	Less often	Neither I nor my Partner suffer from it
Blood poisoning (Sepsis)	◯	◯	◯	◯
Inflammation of the sinuses (par nasal Inflammation)	◯	◯	◯	◯
Urinary tract infections (bladder Inflammation)	◯	◯	◯	◯
Lung infection ◯ (pneumonia)	◯	◯	◯	
Wound infections	◯	◯	◯	◯
Skin and soft tissue (abdominal) infections	◯	◯	◯	◯
Bone and joint infections	◯	◯	◯	◯

other infections (please tell)

123. Compared to your partner or friends of the same age, do you need to take antibiotics more often than they do?

- ◯ Yes
- ◯ No

124. Can you please tell us the names of the medical preparations you have taken?

125. If you still have (a supply of) antibiotics, for how many days did you have to take them?

126. If you still have antibiotics, what were the reason /cause / diagnosis for them? (Here it makes sense to refer to the recent medical. documents you have received from your physician/clinic)

127. Compared to your partner or friends of the same age do need to visit your doctor more often?

- ◯ Yes
- ◯ No

128. As a result of your infection(s), have blood cultures been taken?
(blood cultures = micro biological investigations of the blood for culturing bacteria and other germs in your blood)

- ◯ Yes
- ◯ No
- ◯ I don't know

129. Do you need more hospitalizations (hospital stays) than your partner or friends of same Age?

- ◯ Yes
- ◯ No

130. Have you ever contacted multi resistant germs ('hospital germs') for example: MRSA, ESBL, VRSA, VISA, VRE, NDM-1)?

- ◯ Yes
- ◯ No
- ◯ I don't know

131. If yes, which germ(s) were found? (refer to your medical report

- ◯ MRSA (mehticilin resitant staphylococcus aureus)
- ◯ ESBL (extended spectrum β-lactamase producing germs)
- ◯ VRSA (vancomycin-resistant staphylococcus aureas)
- ◯ VISA (vancomycin intermediary sensible staphylococcus aureus)
- ◯ NDM-1 stems (New Delhi metallo-β-lactamase-1)
- ◯ VRE/ GRE (vancomycin / glykopeptide resistant enterococces)
- ◯ I don't know the name of the germ
- ◯ Sonstiges (bitte angeben)

132. In comparison to your partner or friends of the same age, did you contact these germ(s) more often, or were you frequently in the hospital as a result of contacting these germ(s)?

- ◯ I contacted such a germ(s) more frequently, and was also more frequently in the hospital .
- ◯ I contacted such a germ(s)more frequently but my partner/ friend and I were just as frequent in hospital or my partner/ friend were even more often in hospital.
- ◯ I contacted such a germ(s) less often/ as often as my Partner /friend and I went more frequently to the hospital.
- ◯ I contacted such a germ(s) less frequently/ just as often as my partner/ friend and my partner/ friend was more frequent/ as often as I was in the hospital.
- ◯ Neither I or my partner/ friend ever had such a germ

133. Do you have allergies? (multiple answers possible)

- ☐ Yes, hay fever or asthma (for example, grasses, pollen, dust mites, etc...)
- ☐ Yes, to medications or nutrients (not including lactose intolerance, for example)
- ☐ Yes, to contact allergens (for example nickel , rubber, Latex)
- ☐ No

134. Does your partner or friends of the same age have allergies? (multiple answers possible)

- ☐ Yes, hay fever or asthma (for example, grasses, pollen, dust mites...)
- ☐ Yes, to medications or nutrients (not including intolerances to lactose, for example)
- ☐ Yes, against contact allergens (for example nickel, rubber, latex, etc.)
- ☐ No

Many thanks for your help and participation!

You can press on "back" any time to see your previous answers, again.

135.

The entry of your e-mail address is voluntary and we will also accept your data without the indication of your e-mail address. However, it would be a great help in our research if we could contact you in the case of back queries or further consecutive studies. Please not that when we do contact you, you have the right to take back your personal data, and erase your personal email if you so desire.

When you click on "ready" you will finish this survey and confirm that you agree with the analysis of your data. Thank you!
