# Supplementary material for: Increased Risk for Infections and Allergic Disease in Hereditary Hemorrhagic Telangiectasia
Source: J Clin Med. 2024 Jun 27;13(13):3752. doi: 10.3390/jcm13133752 (PMC11242906; doi:10.3390/jcm13133752)
Supplement: Supplementary file 1 [file jcm-13-03752-s001.zip › Revision_Supplemental material 1_diagnostic assignements.pdf]

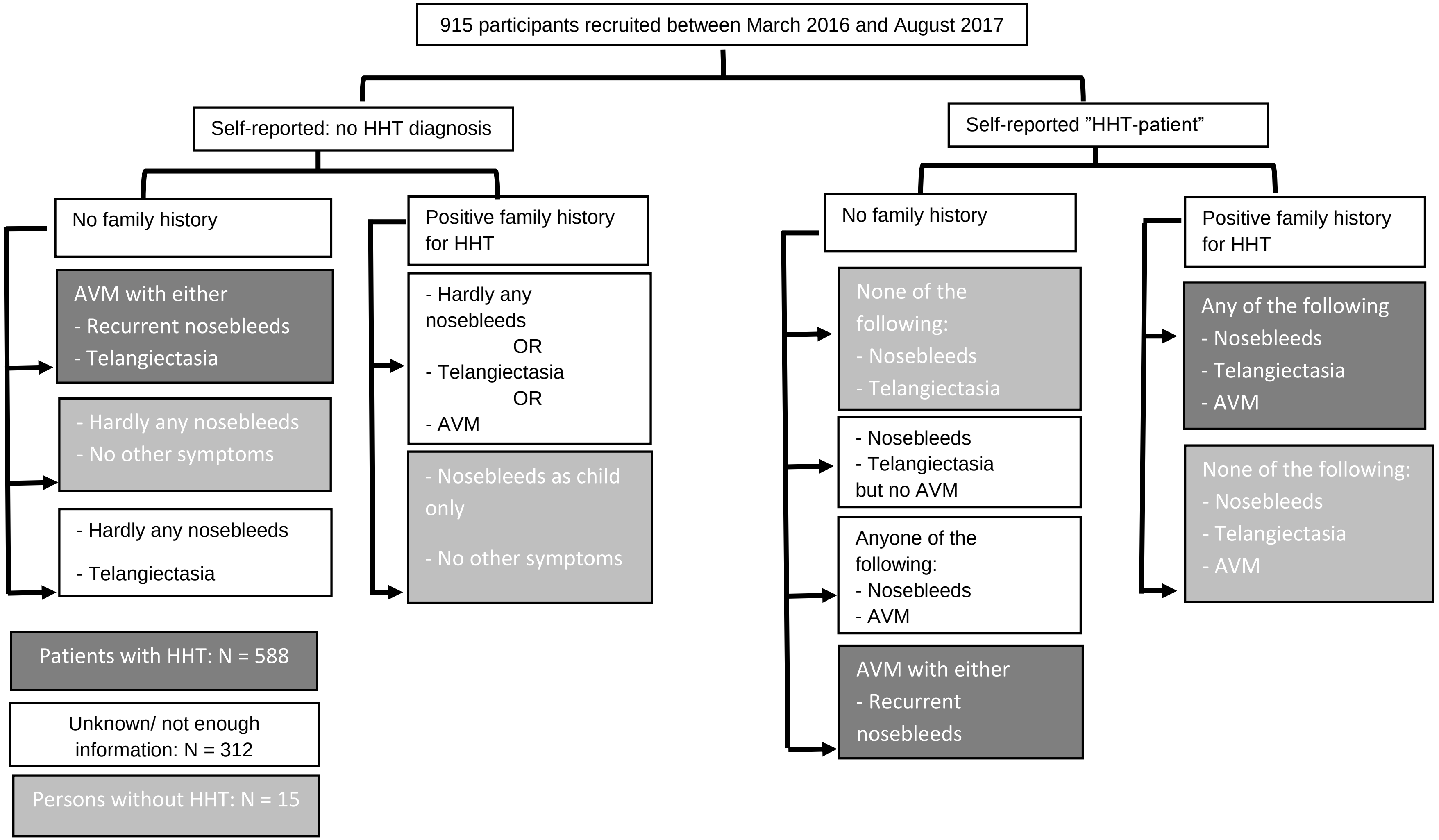

Supplement material 1: Stratification of diagnostic assignments

Similarly to Hosman et al. [16], this flowchart shows the modified Curaçao criteria (positive family history, recurrent epistaxis, multiple telangiectasia, visceral arteriovenous malformation) while using different terms to describe the three groups “Patients with HHT”, “unknown/ not enough information” and “persons without HHT”.

HHT = hereditary hemorrhagic telangiectasia, AVM = arteriovenous malformation (e.g. hepatic/ cerebral/ pulmonary vascular malformation or gastrointestinal involvement), N = number of participants.
